# Comparative Evaluation of Resin Dentin Interface using Universal and Total- Etch Adhesive Systems on Sound and Eroded Dentin: In Vitro Study

**DOI:** 10.1055/s-0041-1734469

**Published:** 2021-10-01

**Authors:** Ghayathri Kanniappan, Padmini Hari, Ravikanth H. Jujare

**Affiliations:** 1 Faculty of Dentistry, MAHSA University, Kuala Lumpur, Malaysia

**Keywords:** confocal laser scanning microscope, eroded dentin, resin tag length, total-etch adhesive, universal adhesive

## Abstract

**Objective** This study aimed to compare the resin-dentin interface of sound and eroded dentin using universal and total-etch adhesive systems.

**Materials and Methods** Forty caries-free extracted human premolars were collected, and the occlusal surfaces were ground by using slow speed diamond disc with copious water supply until a flat superficial dentin was exposed. The test group underwent erosive cycle (n = 20), and another group (n = 20) was reserved for control group. Erosive protocol consisted of immersion in 1.23% citric acid for 1 minute every 12 hours and stored in artificial saliva. Both the control and eroded teeth were further subdivided (n = 10) for composite restoration by using either self-etch or total-etch systems. Then the tooth samples were sectioned longitudinally and observed under confocal laser scanning microscope at ×10 magnification to evaluate resin tag length and hybrid layer thickness.

**Statistical Analysis** The data obtained were analyzed by using independent t-test.

**Results** The highest mean value of the resin tag length and thickness of hybrid layer was observed with total-etch system in sound dentin group compared with other groups (p < 0.001).

**Conclusion** The resin-dentin interface of sound dentin was found to be better than eroded dentin by using total-etch system. The resin-dentin interface of eroded dentin was superior to sound dentin by using self-etch adhesive system.

## Introduction

Qualities of adhesive restorations are determined by three main crucial factors that include condition of tooth, adhesive material properties, and the clinical procedure. In a joint effort to promote the bonding between adhesive system and tooth structure, clinical adhesive system has made a giant leap over the years. Despite having various generations of adhesive system, the most preferred adhesive system are the fifth generation (total-etch) and the seventh generation (self-etching system).

The principle of total-etch technique is to use acid etching for removal of smear layer or smear plug and also expose the collagen matrix, which is followed by a subsequent application of a single bottle self-priming bonding agent that fuses the primer and adhesive component. Presence of any incomplete collagen expansion leads to impaired penetration of the resin and compromise bonding capacity of these systems. The self-etch system may consist of two bottle system, where the first bottle is the combination of acid and primer and the second bottle is the bonding agent. Another variation of the self-etch adhesive system is the all-in-one bottle system. 1

Self-etch adhesive systems are superior to total-etch systems due to the reduction of possibilities of iatrogenic induced clinical mishaps throughout acid conditioning, rinsing, and drying, which are more common in total-etch systems. 2 On the contrary, self-etch system may fail to etch the enamel surface as efficiently as phosphoric acid, in achieving depth of penetration into the tubules. 3 4

The modern dentistry has observed development of various generations of bonding agents in the evolution of adhesive dentistry over the decades. Invention of universal adhesive can be considered as a great milestone in adhesive dentistry. It is also known as “multipurpose” or “multimode” adhesive due to its flexible nature, which can be used as either total-etch or self-etch technique. 5 6

However, it is challenging to use the same bottle of adhesive for all types of tooth structure such as sound, carious, attrited, eroded, or any other noncarious lesions as the nature of the tooth substrate varies according to the tooth condition. 3 Although the focus is more on difference between sound or carious lesions, the noncarious lesions especially erosive tooth wear is also a common clinical finding. Increased incidence of teeth erosion is reported in adults and children due to current diet, lifestyle, oral hygiene habits, eating disorders, or gastrointestinal diseases. 7 8 9 10 The common findings are erosion involving the maxillary anterior palatal surfaces that affects patient’s aesthetics, functional occlusal efficiency, and also incisal guidance. 8

The depth of tooth erosion depends on the amount of dentin exposure. Moderate-to-severe erosive tooth wear gives a significant clinical challenge for dental practitioners in terms of complexity of treatment and longevity of the restoration. The treatment modality for tooth erosion initially was focused more on invasive procedures such as indirect all-ceramic restorations, resin-bonded palatal metal alloy veneers, but currently the focus has shifted to a minimally invasive dentistry like direct composite restorations. 11 12 Of late, self-adhering flowable resin composites were introduced eliminating the prerequisite of application of etch and bond. 13 However, various authors have reported more adhesive failures compared with cohesive failures in eroded teeth. 14 15

The adhesive techniques are critical, and the success of direct restorations is impeded by inadequate adhesion and hybrid layer degradation. Nowadays, researchers are focusing on the development of surface treatments to enhance adhesion or a decrease in hybrid layer degradation. Gabriela et al reported that arginine dentine pretreatment had positive effect on adhesion especially with self-etch adhesive system. 16

From the reported studies, it was observed that eroded dentin exhibited lower bond strength compared with sound dentin, but this might also be affected by the age factor. A clinical based study conducted by Federlin et al has showed that class V restorations bonded to the unprepared surface of eroded dentin for a period of 12 months, displayed good retention regardless of the type of restoration. 17 However, another similar study reported high rates of restoration failures up to 44 to 50%. 18 The effect of mode of adhesion and chlorhexidine on the microtensile strength of a universal bonding agent to sound and caries-affected dentins reported by Lima et al showed that the microtensile bond strength had no significant difference between sound dentin and caries-affected dentin. 19

According to Yabuki et al, bonding in eroded enamel was observed to be lower than in sound enamel. Bond strength of the restoration of eroded dentin are being questioned in day-to-day practice, and only few reports explained the relationship between the bond strength and the restoration especially using universal adhesive system. 20

Thus, there is a paucity of evidence that explains the relationship between total-etch and self-etch technique, especially on eroded dentin using universal adhesive system. Additionally, interfacial microscopic examination of resin dentin interface is conventionally done by using optical microscope and SEM. 21 However, CLSM has emerged as a better nondestructive reliable tool to assess the subsurface characteristics of resin dentin interface. 22 23

Hence, the aim of this study was to compare the resin-dentin interface of normal and eroded dentin by using universal adhesive system and total-etch adhesive system with the aid of confocal laser scanning microscope (CLSM). The alternate research hypothesis was the resin-dentin interface of sound dentin is better than eroded dentin by using universal adhesive system, and formation of the resin tag and hybrid layer in total-etch system is longer and thicker compared with self-etch system in eroded dentin.

## Materials and Methods

### Experimental Design

A randomized in vitro controlled study was conducted. Study protocol was submitted to Research management Centre, MAHSA University, and the ethical approval was obtained. 24

Forty caries-free human premolars extracted for the purpose of orthodontic treatment were collected and sterilized according to guidelines published in Occupational Safety and Health Administration and the Centre for Disease Control and Prevention. 23

### Sample Size Estimation

Sample size was estimated based on two means formula. The sampling was performed by G Power Software. Estimated sample size is 10 per group and 40 samples in total. 23

### Sample Preparation

**Fig. 1:**
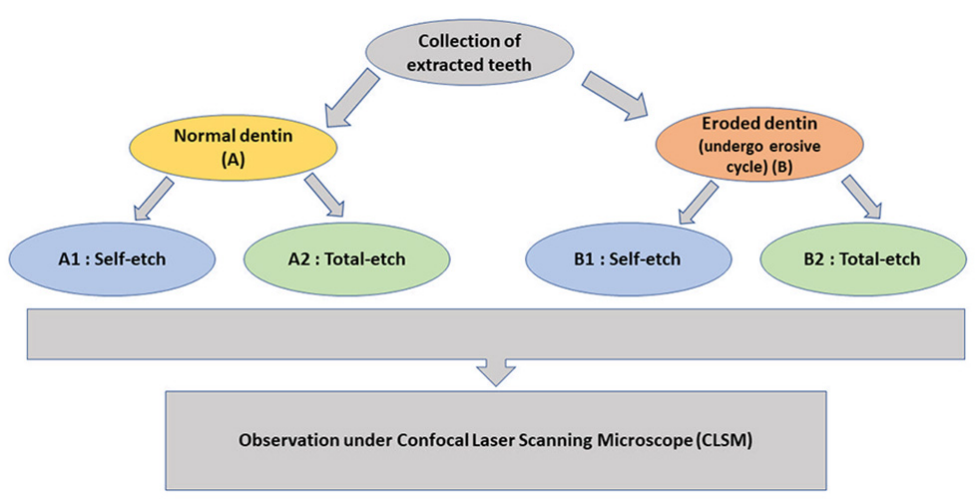
Flow chart of the methodology.

All the teeth samples were mounted in wax block, and the occlusal surfaces of the teeth were ground by using slow speed diamond disc with copious water supply until all the occlusal enamel layer was removed and a flat section of superficial dentin was exposed. The teeth were then randomly divided into two groups of 20 samples each. Control group A representing sound dentin (20 teeth) and test group-B representing eroded dentin (20 teeth), respectively. Ten teeth from each group were further randomly subdivided into subgroups A1 and B1 representing total-etch system, and A2 and B2 representing self-etch system, respectively ( Fig. 1 ). After the sectioning is completed, all the samples were stored in S artificial saliva in respective labeled containers till further use at 37°C. The composition of the artificial saliva used in this study were KCl (16.1 mM), NaCl (14.4 mM), K _2_ HPO _4_ (2.0-mM), MgCl _2_ •6H _2_ O (0.3 mM), CaCl _2_ •2H _2_ O (1.0 mM), and sodium carboxymethyl cellulose (0.10 g% ; CMC-Na) at pH of 7.0. 20

Selection of teeth and division of teeth was done by another investigator to avoid the selection bias by the operator.

### Erosive Protocol for the Test Group Samples (B1 and B2)

The teeth in test group underwent erosive protocol that is similar to the study conducted by Yabuki et al, which involves immersion of test group teeth (eroded dentin) in 1.23% citric acid solution (pH = 2.1, 6.4 × 102 mol/L) for 1 minute every 12 hours followed by incubating in artificial saliva at 37°C. This cycle was repeated for five times in 3 days. 20

In the control group, the sound dentin sample teeth were simply incubated in artificial saliva at 37°C for 3 days without exposure to erosive cycle.

### Fluorescent Labeling of Bonding Agent

Rhodamine B dye with 0.01%wt mixed with 5 mL of universal adhesive agent (G-Premio Bond, GC Corporation, Tokyo, Japan) as suggested by Júnior et al was used to obtain an intense fluorescence to aid in the visualization of the bonding agent distribution on the resin-dentin interface. 25 The materials used in this study are specified in Table 1 .

**Table 1:** Materials used for the study

Trade name	Material details	Manufacturer	Lot no
FineEtch	37% Phosphoric acid	Spident Co., Ltd	FE18240
G-Premio Bond	Universal adhesive system	GC Corporation, Tokyo, Japan	1712092
G-ænial Universal Flo	Flowable composite restorative material	GC Corporation, Tokyo, Japan	1806182

### Restorative Procedure

**Fig. 2:**
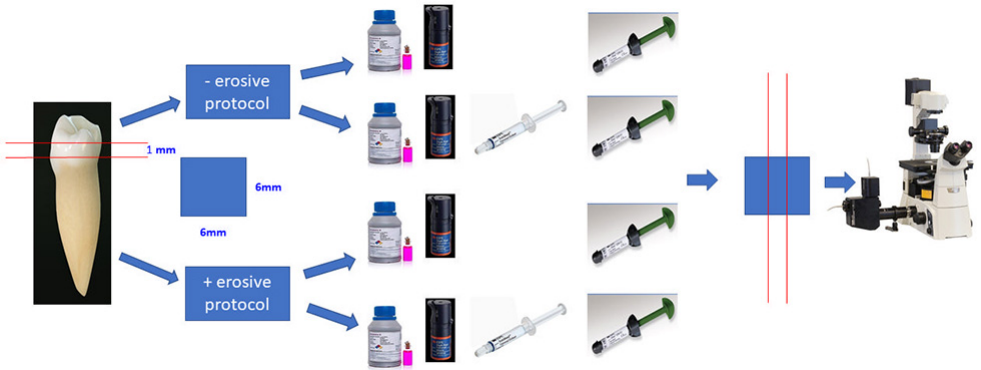
Flow chart of the restorative procedure.

Following manufacturer’s instructions, the restorative procedure in groups A1 and B1 was performed with total-etch adhesive technique, while A2 and B2 restorative procedures were performed by using self-etch adhesive technique ( Table 2 ). The flow chart of the restorative procedure is shown in Fig. 2 .

**Table 2:** Group description

Group	Description
A1	Sound dentin, total-etch
A2	Sound dentin, self-etch
B1	Eroded dentin, total-etch
B2	Eroded dentin, self-etch

#### Total-Etch Adhesive Technique

In total-etch adhesive technique, A1 and B1 exposed dentin surface of each tooth was treated with 37% phosphoric acid gel for 20 seconds, then rinsed with water for 5 seconds, and gently dried using three-way syringe with gentle airstream for 5 seconds. The universal adhesive agent (G-Premio Bond, GC Corporation, Tokyo, Japan) was then applied uniformly on the tooth occlusal surface for 10 seconds with a disposable applicator tip, dried with gentle airstream for 5 seconds at a 3-cm distance to evaporate the solvent, and light cured for 10 seconds using a LED device with a power output of 600 Mw/cm ^2^ . Composite build up was done by using a flowable nanocomposite (G-ænial Universal Flo, GC Corporation) until a 2-mm occlusal height was achieved. Siqveland wide matrix band and retainer was placed around the tooth followed by occlusal restoration. Once the top surface of the restoration was light cured for 20 seconds, the matrix band was removed and again light cured from all four sides for 20 seconds to ensure adequate curing of the restoration. The tooth samples were placed in their respective labeled containers and incubated in artificial saliva at 37°C.

#### Self-Etch Adhesive Technique.

In self-etch adhesive group, the A2 and B2 exposed dentin surface of each tooth were dried by gentle airstream using three-way syringe, and then the universal adhesive agent was applied on the tooth occlusal surface for 10 seconds with a disposable applicator tip, dried with gentle airstream for 5 seconds at a 3-cm distance to evaporate the solvent, and light cured for 10 seconds using a LED device with a power output of 600 Mw/cm ^2^ . This was followed by composite restoration as explained in 2.6.1.

### Assessment of Resin-Dentin Interface

**Fig. 3:**
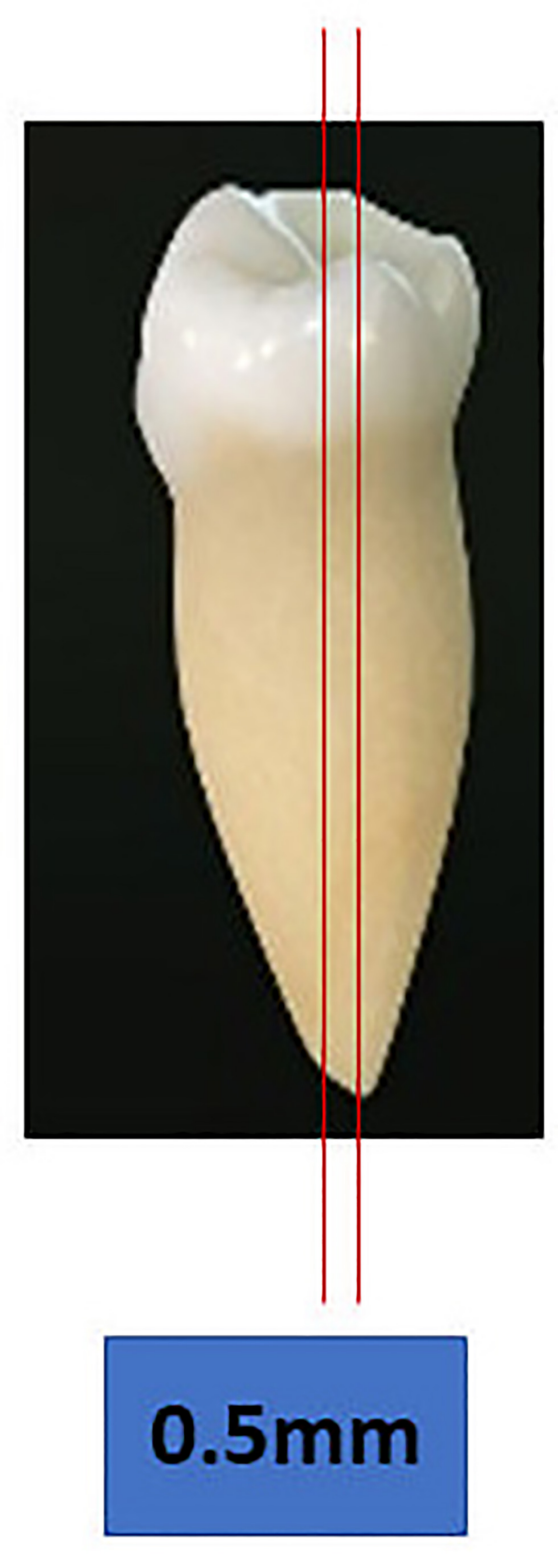
Longitudinal sectioning of the tooth. The thickness of the longitudinal sectioning of the tooth should be 0.5 mm thickness.

After completion of the restorative procedures, two longitudinal sections were made along the long axis of the teeth in the middle portion of the tooth to obtain a tooth section measuring approximately 0.5 mm thickness using a slow speed diamond disc under copious water supply ( Fig. 3 ). The sectioned samples were further grinded on a carborundum stone to achieve a smooth uniform, even surface. The samples were pat dried with a paper towel and mounted on the glass slide with a coverslip.

**Fig. 4:**
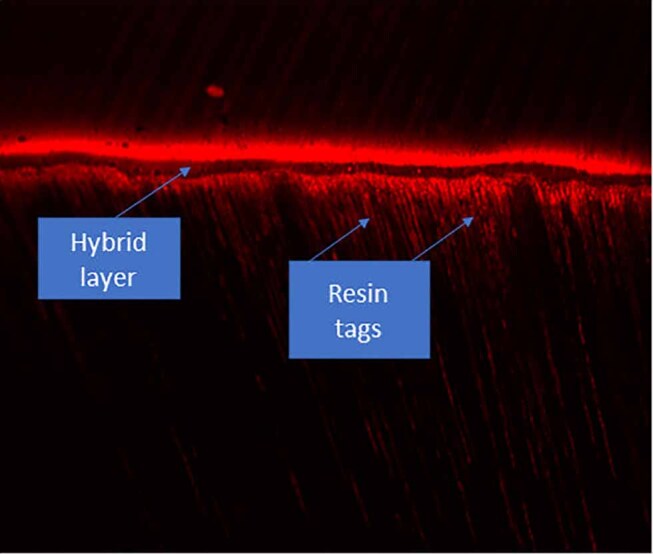
Confocal laser scanning microscope image of resin-dentin interface. Confocal images using a ×10 objective, Argon laser illumination at 50% intensity using a 514 nm excitation wavelength. Confocal slits were set at 25 µm with a 536 nm long-pass filter.

The resin-dentin interface of the tooth samples in both test and control groups was examined to observe resin tag length and hybrid layer thickness by using CLSM (LSM 5 Pascal Exciter, Zeiss, Germany) under ×10 magnification and Argon laser illumination at 50% intensity with 514 nm excitation wavelength. Confocal slits were set at 25 μm with a 536 nm long-pass filter ( Fig. 4 ).

The images were analyzed and the average of three readings was recorded for each parameter. The resin tag length and hybrid layer thickness were measured by using Image Browser Software (Zeiss, Germany) in micrometer by a single trained examiner. Prior to the experimental study, intraexaminer calibration was done and the reliability was verified at two different time intervals in two weeks gap. Intraclass correlation coefficient (ICC) reliability index value was estimated to be greater than 0.9.

### Statistical Analysis

The data obtained was tabulated and analyzed by using independent *t* -test.

## Results

**Fig. 5:**
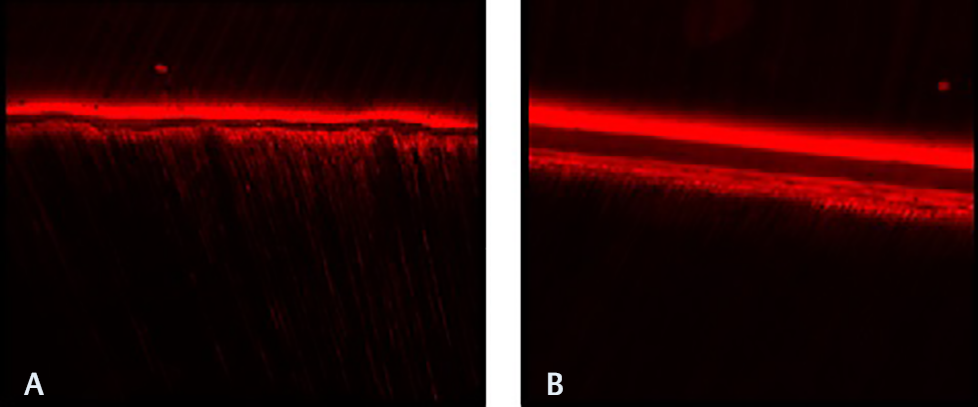
Confocal laser scanning microscope images of ( **A** ) total-etch versus ( **B** ) self-etch in sound dentin. Total-etch system in sound dentin displays longer resin tags and thicker hybrid layer compared self-etch system in sound dentin.

**Fig. 6:**
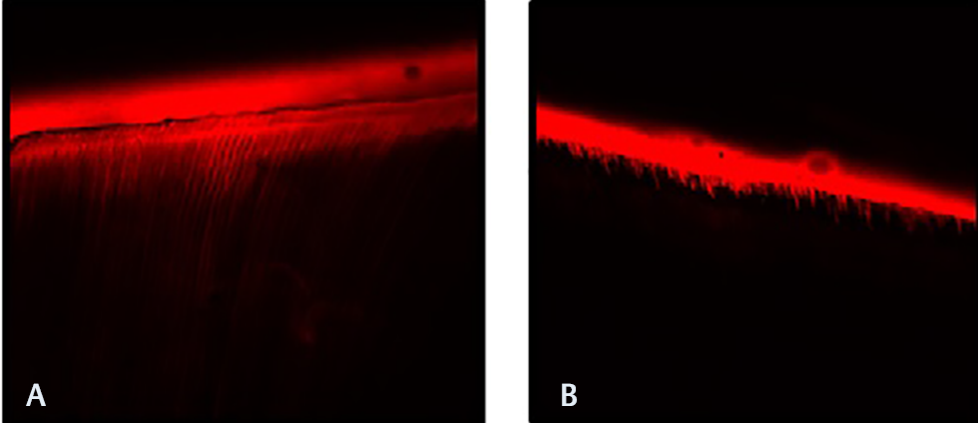
Confocal laser scanning microscope images of ( **A** ) total-etch versus ( **B** ) self-etch in eroded dentin. Total-etch system in eroded dentin displays longer resin tags and thicker hybrid layer compared with self-etch system in eroded dentin.

Resin dentin interface as observed under confocal microscopy was evaluated in this in vitro study. Resin tag length and hybrid layer thickness were studied to assess the difference between sound and eroded dentin by using total-etch and self-etch adhesive systems. The mean values of the resin tag length and thickness of hybrid layer for all the groups were tabulated in Tables 3 and 4 , respectively. Both the normal and eroded dentin exhibited formation of resin tags and hybrid layer. The confocal images of sound and eroded dentin using total-etch and self-etch system are depicted in Figs. 5 and 6 , respectively.

**Table 3:** Mean ± standard deviation of resin tag length in sound and eroded dentin

	Sound dentin	Eroded dentin	*p* -Value
Total-etch system	104.94 ± 6.27	82.52 ± 3.9	*p* ≤ 0.001 ^**^
Self-etch system	19.6 ± 2.11	27.01 ± 3.28	*p* ≤ 0.001 ^**^
*p* -Value	*p* ≤ 0.001 ^**^	*p* ≤ 0.001 ^**^	

**Table 4:** Mean ± standard deviation of hybrid layer thickness in sound and eroded dentin

	Sound dentin	Eroded dentin	*p* -Value
Total-etch system	6.71 ± 0.41	5.45 ± 0.25	*p* ≤ 0.001 ^**^
Self-etch system	2.36 ± 0.22	3.61 ± 0.48	*p* ≤ 0.001 ^**^
*p* -Value	*p* ≤ 0.001 ^**^	*p* ≤ 0.001 ^**^	

### Resin Tag Length

With total-etch adhesive system, the sound dentin was superior to eroded dentin with longer resin tag length which has a mean length of 104.94 ± 6.27 µm while the eroded dentin has a mean length of 82.52 ± 3.9 µm, and the difference was statistically significant ( *p* ≤ 0.001). In contrast to the above, with self-etch system, the mean resin tag length was significantly greater in eroded dentin (27.01 ± 3.28 µm) than that of the sound dentin (19.6 ± 2.11 µm) at *p* ≤0.001. When the total-etch and self-etch adhesive systems were compared, the resin tag length was longer in total-etch adhesive system compared with self-etch adhesive system irrespective of sound or eroded dentin and was statistically significant ( *p* ≤ 0.001).

### Hybrid Layer Thickness

Using total-etch adhesive system, the hybrid layer thickness observed was better in sound dentin with a mean thickness of 6.71 ± 0.41 µm than in eroded dentin with mean thickness of 5.45 ± 0.25 µm. This difference was statistically significant ( *p* ≤ 0.001). In contrast, the hybrid layer showed a significantly greater thickness in eroded dentin than in sound dentin (3.61 ± 0.48 vs. 2.36 ± 0.22 µm, respectively) with self-etch adhesive ( *p* ≤ 0.001). The hybrid layer thickness produced by total-etch adhesive system were thicker compared with self-etch adhesive system. The difference of hybrid layer thickness produced in sound and eroded dentin using total-etch adhesive and self-etch adhesive system were statistically significant.

## Discussion

In this in vitro confocal study, the resin tag length and hybrid layer thickness were analyzed at the resin dentin interface. Extracted human teeth were used as the samples in this study to mimic in vivo situation closely to evaluate bond strength. All 40 extracted tooth samples were stored in 10% formalin for 14 days as per Center for Disease Control and Prevention recommendation. This is to prevent the risk of cross infection as extracted teeth are considered as a potential source of blood borne pathogens(OSHA).

The extracted teeth were ground superficially on a carborundum stone with water as lubricant until the complete section of occlusal dentin was exposed. Selection of dentin layer is an important factor to be considered in the study design, as it was reported that different layers of dentin display different level of bond strength. Dentin layer can be classified as superficial, middle, or deep dentin. Bond strength of dentin bonding agent decreases as the depth of the dentin increases. Pegado et al also evaluated the microtensile bond strength to deep and superficial permanent dentin using different bonding agents, and concluded that the bond strength obtained in superficial dentin was significantly higher than in deep dentin for all adhesive systems tested. 26

The usage of superficial dentin for maximum bond strength in this study was validated by McCabe et al. 27 The test group samples underwent erosive cycle or pH cycling as described by Yabuki et al and Zimmerli et al to depict an erosive condition. 7 20 The erosive cycle protocol was done by using 1.23% citric acid due to its low pH with low calcium and fluoride concentration. 28 Citric acid also simulates acidic drinks, which is a prime cause for dental erosion. Although in vitro erosive protocol may not depict the exact oral clinical environment, alternate exposures to short cycles of acid, followed by immediate immersion into artificial saliva can simulate demineralization and remineralization cycle as seen in oral cavity. 29 The pH of the citric acid used in this study was 2.1 to mimic the pH of the regular acidic beverages consumed and available in the market. 7

Resin dentin interface is usually evaluated in vitro by using dye penetration studies to detect bond failure at the enamel-resin interface. Confocal laser scanning microscopy (CLSM) is a valuable technique for the visualization of bonding structures such as a hybrid layer in dentin. 30 31 CLSM has gained reputation over the years in evaluation of interfacial microscopic examination of adhesive agents due to its laser light characteristics and minimal aberration and diffraction artifacts as compared light microscopy. 13 CLSM technique requires very low concentration of fluorochromes, which are soluble in the adhesive liquids without altering their properties. 13 23 32

CLSM was chosen in this study as it can generate noninvasive serial optical sectioning of intact specimens eliminating the artifacts arising with manual sectioning as seen in scanning electron microscopy (SEM) and transmission electron microscopy techniques (TEM). 23 CLSM was also reported to render a detailed information than the SEM because of its nondestructive nature and elimination of other artifacts such as shrinking, swelling, or detachments that can happen with SEM. Moreover, CLSM gives the possibility to distinguish the components of bonding agents that can be visualized up to 100 µm below the surface. 22 32 33

To distinguish the resin-dentin interface under confocal microscopy, the universal adhesive agent was incorporated with a concentration of 0.1 mg/mL of Rhodamine B dye before application on the dentin surface, as it easily penetrates into the dentin and act as a contrast agent. 25 34

Depending on the type of group, the restorative procedures were done on all the samples. Resin-dentin interface, has a thin layer of resin-reinforced dentin that binds the two different materials together on a molecular level which is often called as hybrid layer. It prevents leakage and develops high resistance to acidic medium. The penetration of adhesive agent into the dentin tubules forms the resin tag. In conventional total-etch system, acidic pretreatment removes the smear layer covering the dentin thus exposing the dentin tubules.

Upon application of bonding agent, it spreads on the dentinal layer and penetrates into the dentin tubules, thus forming the resin tags. In self-etch system, acid is already incorporated (all-in-one bottle system) to remove the smear layer. However, the removal of the smear layer is inadequate and is much lesser compared with total-etch system.

The restored teeth were ground to 0.5 mm thickness, and the middle portion of the tooth were taken and fixed on a glass slide and coverslip to assess the parameters under the CLSM. CLSM aids in distinguishing and measurement of the hybrid layer and resin tags. 23 The average of three readings per sample were recorded to minimize the human errors.

In the present study, it was observed that the total-etch system had better permeability compared with self-etch system in both sound and eroded dentin. This observation can be explained based on the following reasons:

- The lower pH of phosphoric acid in total-etch adhesive system serves as a strong etchant which could have led to demineralization and increased permeability of the dentin. 23
- The total-etch technique is a moist bonding technique. It increases the resin tag formation by preventing the collapse of the collagen fibrils by maintaining the collagen fibril separation. 18

In self-etch adhesive system, there is no rinsing involved. Thus, the lower pH of self-etch adhesive leads to formation of dentin by-products, which limits the level of demineralization and penetration to the superficial layer of the dentin. Additionally, the smear layer contains mineral components that may neutralize the acidic component of the self-etch adhesive, thereby affecting the demineralization of the tooth surface. 35

Present study results were in agreement with other studies done on sound dentin, where in total-etch system was shown to have a better bonding capacity compared with self-etch adhesives by using various analytic techniques. 1 13 36 37 However, bonding in eroded teeth is a great challenge. Augusto et al and Milosevic et al have reported more adhesive failure than cohesive failure in eroded dentin which clearly indicates failure in bonding agent. In erosive teeth, due to structural dissolution, the erosion leads to mineral loss and exposure of the collagen fibrils and also dentin tubules. 14 15 36 Emergence of universal adhesive system may possibly offer a solution for bonding in eroded teeth.

In the present study, the difference in bonding ability of eroded and sound dentin was assessed with two different adhesive systems. It was observed that total-etch system had greater length of resin tags and hybrid layer thickness in sound dentin compared with eroded dentin. This could be due to the difference in the mineral content of the tooth. The erosive cycle subjects the tooth to acidic medium which demineralizes the tooth surface leading to mineral loss and collapse of the collagen meshwork. Thus, it limits the penetration of the bonding agent into the dentin tubules despite having acid etching using 37% phosphoric acid, which removes the smear layer. 20

However, with self-etch adhesive system, the resin tag length and thickness of hybrid layer were more pronounced in the eroded dentin compared with sound dentin. This could be due to incomplete removal of smear layer using self-etch adhesive system in sound dentin. Erosive cycle using acidic medium causes as the tooth surface to undergo some amount of acidic pretreatment leading to demineralization of dentin and removal of smear layer. This finding corresponds to the scanning electron microscope observation by Augusto et al wherein eroded dentin had etched pattern of tooth surface. 15

Laser scanning microscope study by Yabuki et al also confirmed that eroded teeth demonstrated irregular surface patterns which depicts erosion and etching pattern on the dentin. 20 This, explains the cause of greater penetration of resin tags into the dentin tubules of eroded dentin compared with sound dentin, where the demineralization and acid exposure was limited.

## Limitations of the Study

Oral environment related studies always aim to simulate the oral condition as much as possible as it is considered as the ultimate testing environment to predict the restoration behavior. However, oral environment is complex and biodiverse in nature. Thus, in vitro models hold a high significance in providing a consistent environment of oral cavity and aids in studying product degradation in intraoral condition. 38

In this in vitro study, the teeth collected were stored in artificial saliva to simulate the oral cavity fluid and placed in incubator at 37°C to simulate body temperature. The scope of this research was limited to superficial dentin where erosion may involve deeper layers of dentin depending on the severity.

## Future Recommendations

Further studies involving the bond strength to middle and deep dentin layers may be beneficial to understand bonding in these dentin layers. Additionally, long-term observations from in vivo study in a controlled environment will be more ideal to predict the longevity of composite restoration on eroded teeth. This study can also be extended to compare resin-dentin interface of various commercially available universal adhesive agents on eroded dentin.

## Conclusion

The findings of the present research can be summarized as follows:

- Both sound and eroded dentin displayed greater resin tag length and thickness of hybrid layer in total-etch system than self-etch system.
- In total-etch system, sound dentin displayed greater resin tag length and thickness of hybrid layer compared with eroded dentin.
- In self-etch system, eroded dentin displayed increased resin tag length and thickness of hybrid layer compared with sound dentin.

Within the limitations of the present study, the resin-dentin interface of sound dentin was superior to eroded dentin by using total-etch system as it presented longer resin tags and thick hybrid layer. Thus, we failed to reject the alternate hypothesis. Contrary to the above, the resin tag length and hybrid layer thickness observed in self-etch system was longer and thicker in eroded dentin than with the use of total-etch system. Thus, we accept reject the second alternate hypothesis.
